# Local order in a disordered Zintl phase boosts its thermoelectric performance

**DOI:** 10.1038/s42004-021-00593-0

**Published:** 2021-11-03

**Authors:** Teresa S. Ortner

**Affiliations:** Communications Chemistry, https://www.nature.com/commschem

## Abstract

Understanding heat conduction in solids is fundamental to the design of high-performing thermoelectric materials. Now, a combined computational and experimental study shows how local order paired with long-range disorder leads to a desirable reduction in thermal conductivity while retaining high electron mobility in the Zintl phase Eu_2_ZnSb_2_.

High electrical as well as low thermal conductivity are needed for a material to achieve good thermoelectric performance. However, identifying mechanisms that allow for a reduction in thermal conductivity without a commensurate reduction in electrical conductivity remains a challenge. Now, an international team led by Yue Chen, University of Hong Kong, David Singh, University of Missouri, and Qian Zhang, Harbin Institute of Technology, identify local order combined with long-range disorder as a key feature for high-performance thermoelectrics (10.1038/s41467-021-25483-w)^1^.

Materials designed to have low phonon transport properties generally yield low thermal conductivities only at high temperatures, where the effects of anharmonic phonon scattering are strongest. Disorder-based approaches can lower the thermal conductivity also at lower temperatures, but often at the cost of electronic conductivity. The Zintl phase Eu_2_ZnSb_2_ has surprisingly low thermal conductivity over temperatures from 300 to 800 K^2^. The structure’s intrinsic phonon-phonon interactions with rattling behavior, a low bulk modulus, and scattering resulting from a disordered Zn sublattice are the proposed causes for this unusual property. At the same time, unlike in other phases with high degrees of random disorder, Eu_2_ZnSb_2_ maintains good carrier mobility. The structural causes for this, however, had not been fully conclusive.

Previously, Qian Zhang and colleagues had reported that Eu_2_ZnSb_2_ exhibits an intrinsically ultralow thermal conductivity combined with a high carrier mobility of ~50 cm^2^ V^−1^ s^−1^, leading to a high thermoelectric figure of merit *zT* of ~1.0 at 823 K^2^. Now, comparing experimental measurements and first principles calculations of the structure’s thermal conductivity, they were able to rationalize the observed properties. “For our ab initio molecular dynamics calculations, we used a zig-zag model supercell of 80 atoms of Eu_2_ZnSb_2_ to determine the harmonic and anharmonic interatomic force constants. To then pin down the effects of occupation and disorder on the thermal conductivity, we compared the results to another 96 atom supercell model of the fully-occupied Zintl phase EuAgSb,” explains Zhang. They found that locally ordered zinc atoms retain the material’s high electronic conductivity, while long-range disorder keeps the thermal conductivity low. “We knew that the key feature of Eu_2_ZnSb_2_ is a crystal structure that has a Zn-site with a 50% occupancy^3^,” says Zhang, “but the exact crystal model is actually unknown so the high degree of local order was an unexpected find.”

The researchers are now looking to reproduce this finding in related phases. “Nanostructured systems with disorder on one sublattice, combined with a local ordering tendency, could be the key to achieving high thermoelectric performance,” says Zhang.
